# The Influence of Kinesio Taping on the Effects of Physiotherapy in Patients after Laparoscopic Cholecystectomy

**DOI:** 10.1100/2012/948282

**Published:** 2012-05-03

**Authors:** Marcin Krajczy, Katarzyna Bogacz, Jacek Luniewski, Jan Szczegielniak

**Affiliations:** ^1^Physiotherapy Department, Opole University of Technology, 45-271 Opole, Poland; ^2^General Surgery Department, Hospital in Nysa, Nysa, Poland

## Abstract

Physiotherapy in patients after laparoscopic cholecystectomy (CHL) is impeded by postoperative pain which causes a decline in patients' activity, reduces respiratory muscles' function, and affects patients' ability to look after themselves. The objective of this work was to assess the influence of Kinesio Taping (KT) on pain level and the increase in effort tolerance in patients after CHL. The research included 63 patients after CHL. Test group and control group included randomly selected volunteers. Control group consisted of 32 patients (26 females, 6 males), test group consisted of 31 patients (22 females, 9 males). Both groups were subjected to complex physiotherapy, and control group had additional KT applications. Before surgery, during and after physiotherapy, patients were given the following tests: 100-meter walk tests, subjective pain perception assessment, and pain relief medicines intake level assessment. The level of statistical significance for all tests was established at *P* < 0.05. Statistical analysis showed a significant decrease in the time required to cover a 100-meter distance and a decrease in pain perception presented by significantly lower painkillers' intake in the test group in comparison with the control group. The improvement in clinical condition observed in the research indicates the efficiency of KT as a method complementing physiotherapy in patients after laparoscopic cholecystectomy.

## 1. Introduction

Laparoscopic cholecystectomy (CHL) is a type of classic cholecystectomy. Both methods involve removing the gall-bladder containing gallstones. Most frequently, gall-bladder resection is caused by lithiasis or its complications [1–6]. 

Bile ducts surgery is one of the key aspects of general surgery. Surgeries performed within bile ducts come first in terms of the number of carried out surgeries and exceed the number of hernia or appendix surgeries [3–6].

Surgeries within abdominal cavity lead to body function disorders manifested in postoperative paralysis of alimentary canal motoricity. Pain is a significant problem occurring after surgeries [1–6].

The aim of physiotherapy in surgery is to prevent postoperative complications and treatment of postoperative functional disorders. 

Kinesio Taping (KT), which might enhance the effects of rehabilitation, is one of the ways to support physiotherapy after surgeries. Over the last decade, the use of KT has become increasingly common. The tape use in KT demonstrates elasticity and thickness resembling human skin. Kase et al. [7] have proposed several potential effects, depending on the techniques used and degree of tape stretch, such as providing a positional stimulus through the skin, aligning fascial tissues, creating more space by lifting fascia and soft tissue above area of pain/inflammation, providing sensory stimulation, and assisting in the reduction of edema by directing exudates toward a lymph duct.

## 2. Assumptions, Objectives, Hypotheses, and Research Questions

In case of patients after laparoscopic cholecystectomy, physiotherapy is hindered by postoperative pain which limits patients' physical activity and impairs patients' ability to look after themselves. The process of healing of postoperative wound is also accompanied by pain resulting from immobilization after surgery.

The aim of this work was to assess the influence of KT applications on the effects of physiotherapy in patients after CHL. 

KT is one of the ways of supporting physiotherapy after CHL because it might have a positive influence on the stabilization of postoperative wound and reduce pain.

The study was approved by the Ethics Committee of Opole Voivodship (no. 150/2007).

## 3. Material and Methods

The research included 63 patients after CHL treated in General Surgery Department of Hospital in Nysa between April 2007 and August 2008 (Table 1). To achieve study power of 80% with the use of selected statistical tools, and an alpha level established at 0.05, a priori power analysis pointed to the need for a minimum of 30 patients per group. Both test group and control group included randomly selected volunteers who met test qualification criteria. A random-number generator was used to allocate patients to one of the two groups in the study. All patients qualified for physiotherapy gave written informed consent to participate in the research.

Contrastive analysis of parameters recorded before surgery shows that patients from test and control groups did not differ in terms of the level of subjective pain perception, waist size, lung ventilation activity disorders, effort tolerance, and BMI. In both groups, complex physiotherapy was conducted according to standard physiotherapy program. In test group, additional KT applications were used. Scheduled CHL and age between 40–60 qualified patients for the research. Exclusion criteria included lack of patient's informed consent, age below 40 or above 60, respiratory and cardiac failure (NYHA class III-IV), below 40% of FEV1 predicted value, and postoperative complications (e.g., fever).

Before, during, and after physiotherapy, the following tests were given for all patients:

- subjective pain perception assessment (VAS scale),
- 100-metre walk test,
- pain relief medicines intake level.

### 3.1. Subjective Pain Perception Assessment (VAS Scale)

The assessment of pain intensity involved the patient determining the intensity of perceived pain against the VAS scale. It was assumed that 0 represents no pain, 5 represents strong pain perception, and 10 represents maximal pain intensity. The test of subjective pain perception was given one day before the surgery, 24 hours after the surgery as well as on the second, third, and eighth day after CHL.

### 3.2. Effort Tolerance Assessment Based on the 100-Metre Walk Test

The test was carried out along a specially selected straight section of the hospital corridor, always at the same time in the morning. A 50-metre long distance was measured and marked with posts. The starting line was marked with coloured tape. The patient marched along the corridor, back and forth, at the highest possible pace to cover the distance of 100 metres. The patients were informed to adjust the pace of walking to the subjective feeling of tiredness. Running was not allowed during the test. Blood pressure and maximal effort pulse values were recorded. The test was terminated at the moment of the occurrence of intensified feeling of dyspnea or tiredness, increased abdominal area pain, paleness or growing blue, balance disorders, or chest pain [8]. The test was given one day before surgery as well as on the third and eighth day after CHL.

### 3.3. The Assessment of Pain Relief Medicines Intake Level

Pain relief medicines intake level was recorded 24 hours after the surgery and on the second, third, and eighth day after CHL.

The following intake levels and medicine types were defined:

- level 0—the patient felt no pain and did not take painkillers (0 pts.),
- level I—Ketoprofen pain relief 100 mg ampoules were taken max. 3 times a day (1–3 pts.),
- level II—pain relief pump was used intravenously (4 pts.).

In case of no pain perception, the patient did not take any painkilling tablets (level 0). Level I depended on the intensity of pain. Level II of medicines intake was applied when medicines from level I were insufficient and did not ensure patients comfort.

The patients who participated in the research were not prescribed any additional medicines.

### 3.4. Kinesio Taping Applications Used

**Figure 1:**
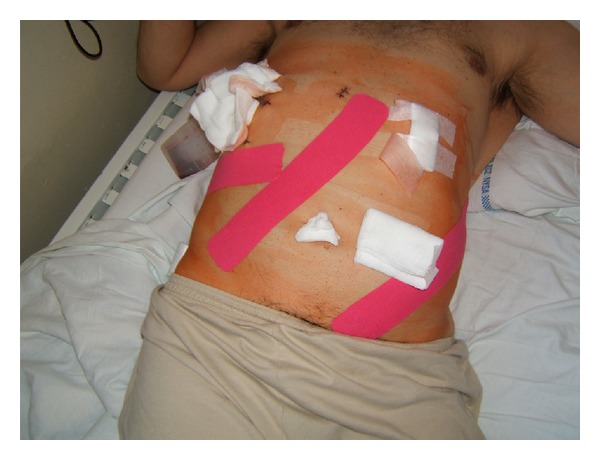
KT application on abdominal muscles and liver fascia.

After excluding contraindications, 24 hours after surgery, patients from the test group were given the following KT applications:

1. muscle application—detoning, applied (from distal to proximal attachment) on external abdominal oblique muscles on the left side;
2. muscle application—detoning, applied on internal abdominal oblique muscles on the right side;
3. fascial application on the liver area (Figure 1).

## 4. Statistical Methods

Statistical analysis was conducted with the use of STATISTICA 7.1 program.

The results were presented as arithmetic average and standard deviation values. Median, quartile, and extreme values were also established (min., max.).

Double classification variance analysis was carried out for dependent variables determining pain level. Depending on the significance of main effects and interactions, the contrastive analysis and the post hoc analysis were also used before and after surgery.

In case of variables with asymmetrical distribution (skewed distribution) differing significantly from normal distribution, nonparametric rank-sum Mann-Whitney *U* Test was used.

Multiple-regression analysis was used for dependent variable defining average medicine intake in particular tests. Statistical significance level was established at *P* < 0.05 for all tests.

## 5. Results

The assessment of subjective pain perception on the VAS scale was carried out before the surgery (*P*) and on the 1st, 2nd, 3rd, and 8th day after CHL.

Statistical analysis showed that the values of subjective pain perception indexes before the surgery (*P*) in case of patients from test and control groups did not differ significantly. The results recorded in consecutive tests showed significantly lower index values in the test group than in the control group. Statistically significant differences were observed between the results of tests carried out on the 1st, 2nd, 3rd and 8th day after CHL in both groups.

**Figure 2:**
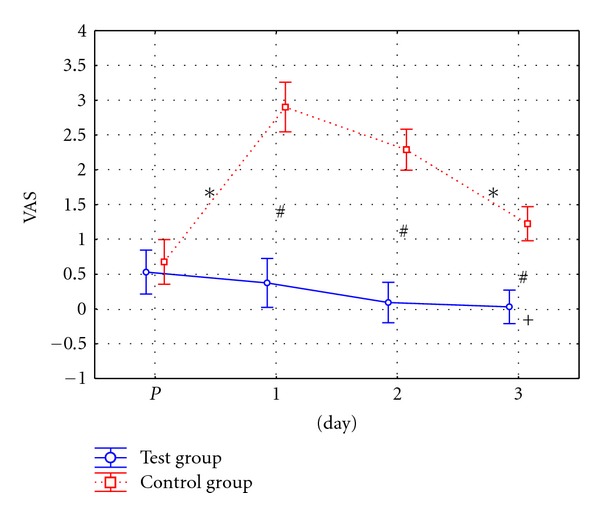
Graphic comparison of pain perception on VAS scale (in pts.) in patients from test and control groups. ∗—statistically significant difference (*P* ≤ 0.05) between consecutive tests. #—statistically significant difference (*P* ≤ 0.05) between groups in particular tests. +—statistically significant difference (*P* ≤ 0.05) in relation to initial test.

Post hoc analysis showed statistically significant increase in the pain level on the first day after surgery in patients from the control group where KT applications were not used. In case of patients from the test group, a decrease in pain level was observed on the first day after surgery. Patients in this group reported a decrease in pain perception level from the initial test to the final test. On the eighth day after surgery, no pain was recorded in this group (Figure 2).

To assess patients' effort tolerance, 100-metre walk test time was recorded before the surgery (*P*), on the third and on the eighth day after the surgery.

Statistical analysis of the results showed that the level of average 100-metre walk test time values recorded before the surgery did not differ significantly between the two groups of patients. In the following tests, 100-metre walk test time was significantly shorter in the test group than in the control group.

**Figure 3:**
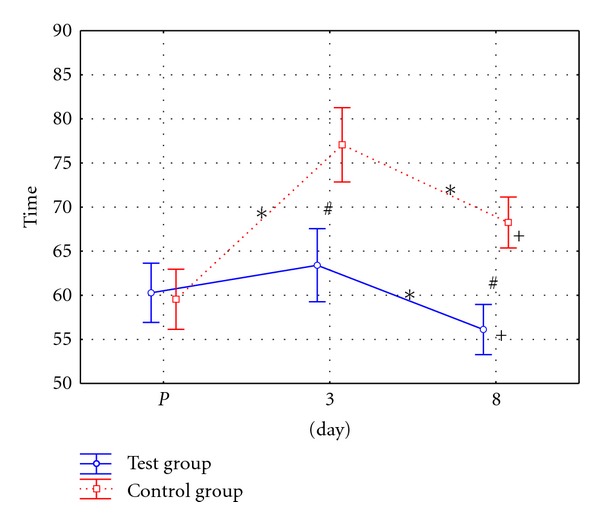
Graphic distribution of 100-metre walk test time (in secs.) in patients from test and control groups.

Post hoc analysis showed significantly longer test time recorded on the third and eighth day in the control group (Figure 3). Significantly lower walk time values were observed in the final test conducted in the test group in comparison with the initial test. At the same time, significantly higher walk test time values were observed in case of patients from the control group.

Patients from test and control groups were given tests on the 1st, 2nd, 3rd, and 8th day after CHL to assess the level of painkilling medicines intake. Because on the 3rd, and 8th days after surgery no consumption of painkillers was recorded, the contrastive analysis was conducted for the results recorded on the 1st and 2nd day after CHL only. 

**Figure 4:**
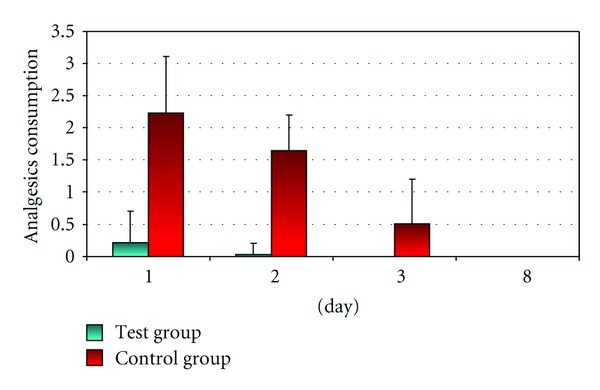
Graphic distribution of average painkillers' intake (in pts.) for patients from test and control group.

Tests conducted on the first and second days after the surgery showed significantly lower level of pain relief medicines intake in the test group than in the control group (Figure 4).

A decreased amount of pain relief medicines was taken by patients in the test group on the second day after CHL. On the third and eighth day after CHL, the need for painkillers was not recorded in this group.

**Figure 5:**
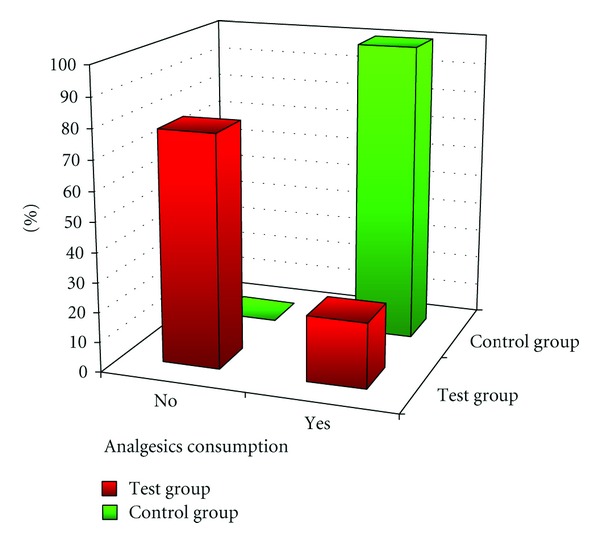
Comparison of pain relief medicines (in %) for patients in both groups.

The research showed that 7 out of 32 (21.88%) patients from the test group took painkilling tablets whereas all of 31 patients (100%) from the control group used pain relief medicines (Figure 5).

Chi-square test analysis showed differences in pain relief medicines intake distribution between patients from the two research groups.

## 6. Discussion

So far, research showed the possibility of using KT in sports medicine and various other medical fields [9–29].

Professional literature reports the possibility of employing KT in surgery. So far, it has proved useful in stabilizing postoperative wounds. There are, however, no reports related to complex physiotherapy treatment with the use of KT in general surgery, including laparoscopic cholecystectomy.

Literature reports no studies analyzing the influence of KT on physiotherapy of patients after surgeries, especially in the area of its influence on the subjective pain perception, effort tolerance or pain relief medicines' intake.

Own research confirms earlier assumption stating that KT is one of the methods which, not only have a positive influence on the stabilization of postoperative wounds, but also has a painkilling effect in case of patients after CHL. It is hypothetical what physiological mechanisms are responsible for the analgesic effects of KT. One possible theory to be taken into account is the gate control theory of pain modulation. The tape has been suggested to stimulate neuromuscular pathways via afferent feedback. Increased afferent stimulus to large-diameter nerve fibers might reduce pain perception level due to an input decrease from the small-diameter nerve fibers conducting nociception. Thelen et al. [30] refer to this theory in their work on KT applications in shoulder pain. Also, González-Iglesias et al. [31] achieve pain-relief effects of KT applications in patients with acute whiplash injury.

Research results confirm the positive influence of KT on the decrease in pain perception resulting in a lower intake of painkilling tablets.

KT applications employed in the physiotherapy process in patients after laparoscopic cholecystectomy had a significant influence on the decrease of pain perception level as well as an increase in effort tolerance and reduced the level of pain relief medicines' consumption in comparison with patients from the control group. It seems that KT, as a complement to complex physiotherapy, might contribute to faster regaining of independence in case of patients after CHL and improve their quality of life.

It seems that KT creates the opportunities for effective support for physiotherapy. Providing postoperative wound stabilization, it allows for reduction of functional activity disorders resulting from surgery and might indirectly enhance the effects of physiotherapy within the short time of hospital treatment. Standard physiotherapy combined with KT might shorten hospitalization time for these patients. Because it significantly decreases painkilling medicines intake and shortens hospitalization time, it might contribute to the reduction of treatment costs of patients' surgeries.

## 7. Conclusions

1. Research showed that Kinesio Taping employed in physiotherapy of patients after laparoscopic cholecystectomy leads to a decrease in pain perception and significantly reduces pain relief medicines' intake.
2. Improvement in effort tolerance achieved in the research shows the efficiency of Kinesio Taping employed for physiotherapy of patients after laparoscopic cholecystectomy.
3. Kinesio Taping provides effective support for physiotherapy and, through postoperative wound stabilization, reduces functional activity disorders resulting from CHL allowing for shortening of hospitalization time.

## Figures and Tables

**Table 1:** Basic characteristic of patients in both groups.

Patients				Age			
Group	No	Female	Male	$\overset{®}{x}$	Min	Max	SD
Test	32	26	6	52.21	40	60	5.97
Control	31	22	9	49.93	40	60	6.55
